# Methodology for estimation of undeformed thickness of arterial tissues

**DOI:** 10.1038/s41598-023-28871-y

**Published:** 2023-02-16

**Authors:** David Schwarz, Jiri Fleisman, Radek Vitasek, Stanislav Polzer

**Affiliations:** Department of Applied Mechanics, VSB-Technical University of Ostrava, 17. Listopadu 2172/15, 708 00 Ostrava-Poruba, Czech Republic

**Keywords:** Mechanical engineering, Soft materials, Mechanical properties

## Abstract

Soft tissue sample thickness measurement is one of the major sources of differences between mechanical responses published by different groups. New method for the estimation of unloaded sample thickness of soft tissues is proposed in this study. Ten 30 × 30 mm and ten 20 × 20 mm samples of porcine anterior thoracic aortas were loaded by gradually increased radial force. Their deformed thickness was then recorded in order to generate a pressure-thickness response. Next, the limit pressure to which the response can be considered linear was estimated. Line was fitted to the linear part of the curve and extrapolated towards zero pressure to estimate unloaded thickness (7 kPa fit). For comparison, data near zero pressure were fitted separately and extrapolated towards zero (Near Zero fit). The limit pressure for the linearity of the response was around 7 kPa. The Unloaded thickness for 30 × 30 mm samples was 2.68 ± 0.31 mm and 2.68 ± 0.3 mm for Near Zero fit and 7 kPa fit, respectively. The Unloaded thickness for 20 × 20 mm samples was 2.60 ± 0.35 mm and 2.59 ± 0.35 mm for Near Zero fit and 7 kPa fit, respectively. The median of thickness difference between smaller and larger samples was not found statistically different. Proposed method can estimate unloaded undeformed sample thickness quickly and reliably.

## Introduction

Cardiovascular biomechanics relies on the mechanical properties of various soft tissues such as myocardium^1^, arteries^2^ and veins^3^. Moreover, they also play a critical role in ageing^4,5^ and it helps us understand the pathophysiology of various diseases such as atherosclerosis^6^, abdominal^7,8^ and ascending thoracic aneurysm^9^ as well as coronary arteries^10^. Obtaining the mechanical properties of soft tissues is, however, not a trivial task for several reasons: local inhomogeneity of sample thickness, challenges to obtaining sufficiently large samples^11^, problems with through-thickness material inhomogeneity^12,13^, sample preconditioning^8^, sample clamping^14^ and wall thickness measurement^15^. In particular, the differences in wall thickness estimation can lead to a significant variation of obtained stresses since uncertainty in stress estimation is largely given by uncertainty in wall thickness estimation which can easily be 20%^15^ even more. For example, the mean wall thickness of an aneurysmal wall was estimated as 2.9 mm ± 0.18 mm when measured by laser micrometer^16^, while it was only around 2 mm when measured by caliper^17^ or 1.5 mm when measured by micrometer^18^. Naturally, mechanical and failure properties obtained in these studies are therefore not comparable at all.

The problem is that arterial tissue is very compliant radially (initial radial stiffness is in the order of tens of kPa^19–21^) and even a small force can create significant deformation. Calipers, are operator dependent, while the contactless optical methods often overestimate measured wall thickness^15^. On the contrary, the micrometers and thickness gauge applied defined force on the defined contact surface thus generating defined contact pressure which results in a systematic shift in wall thickness estimation. Various attempts were published on how to deal with this problem. Currently the best tool for soft tissue thickness measurement is suggested thickness gauge having 95% confidence interval of 0.72 mm when measuring arterial samples^15^ while accuracy for other types of soft tissues is even worse^15^.

That is why we focused on this topic and proposed a new method for nondestructive estimating the unloaded thickness of arterial tissue samples needed for its mechanical testing. It should be fast, sufficiently accurate, easy to use and stay out of the tensile test machine which is typically prepared for performing the tensile test the thickness is needed for. This method is based on the simple idea of extrapolating wall thickness measured at two or more known pressures towards a hypothetical zero pressure.

## Methods

A total of ten descending thoracic aortas were collected from pigs obtained from local slaughterhouses. The age of the pigs was around six months and the weight ranged from 105 to 115 kg. The descending thoracic aortas were taken out within one hour of slaughter and immediately frozen at − 18 °C. Then, they were transported to the laboratory, where they were stored in the freezer until the day of the experiment (less than one month because it was shown freezing storage shorter than 1 month has a negligible effect on the mechanical properties of the aortic tissue^22^). It is underlined tissue freezing was used for our convenience and the method applies for fresh tissues or tissues preserved in any other way.

### Tissue preparation

**Figure 1:**
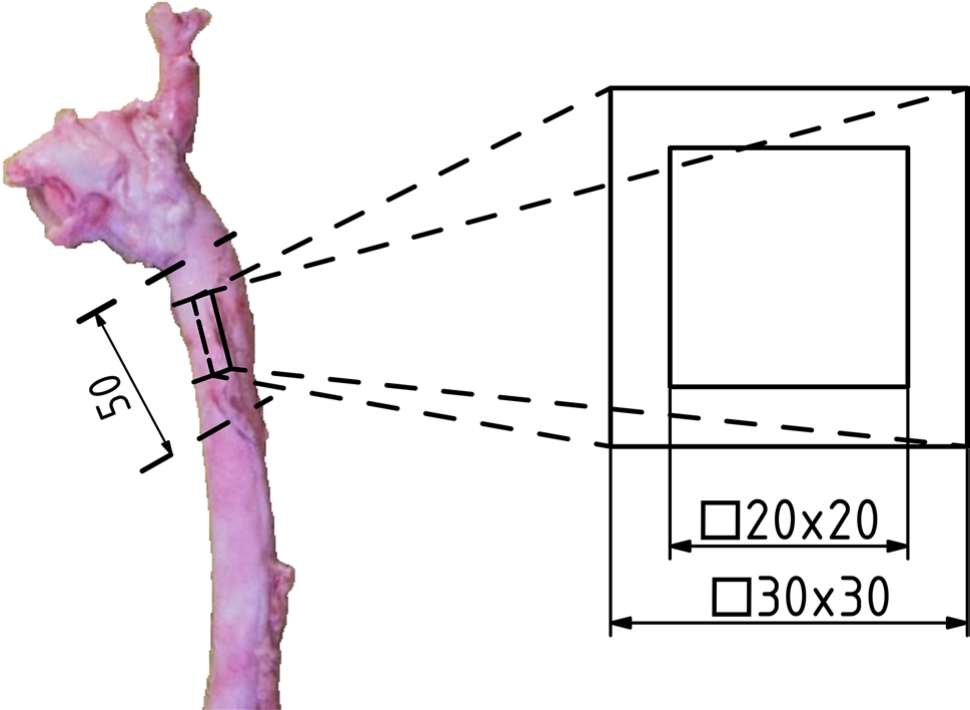
Illustration of place extraction specimen. The 30 × 30 mm large specimen was cut out of each aorta for unloaded thickness estimation. Afterwards 20 × 20 mm sample was cut out of each larger sample for another test.

Deep-frozen specimens of descending thoracic aorta were put in a 5 °C refrigerator for 17 h prior to testing. One hour before testing, the ring of the superior part of the descending thoracic aorta (approximately 50 mm) (see Fig. 1) was extracted from the thoracic aorta. This ring was axially cut in the posterior part of the porcine aorta. Subsequently, all connective tissues and fat was removed. Square samples of 30 × 30 mm were cut from the walls prepared in this way so that one side was parallel to the axis and the other to the circumferential direction. The samples thus prepared were placed in 0.9% NaCl solution at 37 °C for one hour before testing. Each sample was marked accordingly to avoid any confusion later on. Upon completion of the measurement, the 30 × 30 mm specimen was immersed in saline to relax. After relaxation, 20 × 20 mm samples were cut from the 30 × 30 mm samples and the whole measurement process was repeated.

### Experimental setup

**Figure 2:**
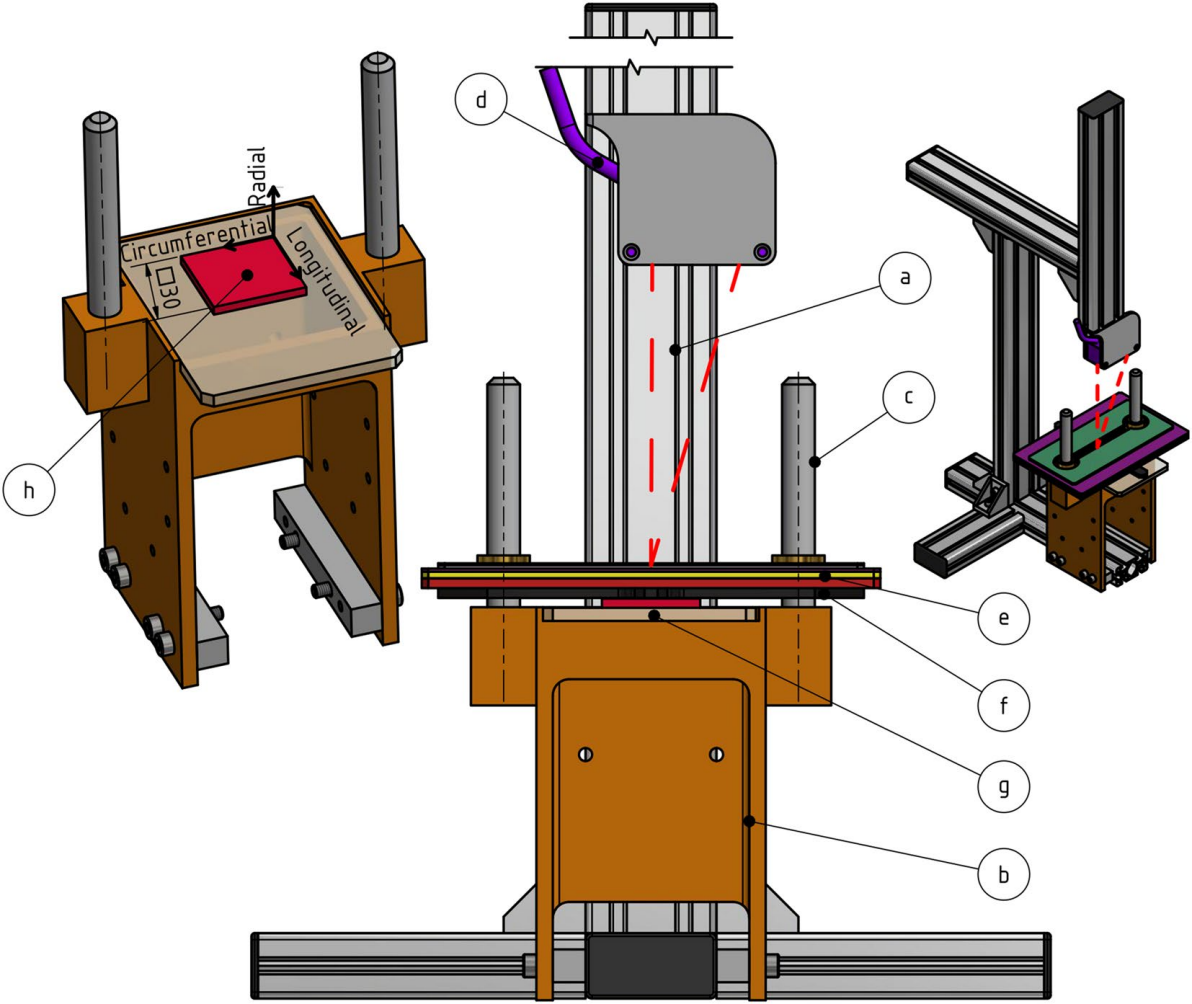
The experimental stand used in the study. It has seven major parts. (**a**) laser holder, (**b**) measuring device body, (**c**) guide pivot, (**d**) laser meter, (**e**) system of weights, (**f**) base weight with slide bearings, (**g**) basic glass. Item (**h**) is the tissue sample.

The measuring device was inspired by a device proposed by Chuong and Fung^21^ who used it for radial compression tests. We propose some modifications, however. The biggest change being that we chose a force driven setup instead of a displacement driven one. That allows the thickness at the measuring location to be measured under constant acting force. The experimental stand consists of seven main parts (Fig. 2). (a) A rigid stand ensuring a fixed position in relation to the body of the measuring machine. (b) The body of the measuring machines is designed so that there is no deformation during loading and therefore no measurement distortion. Further, it contains enough space below the measurement area to accommodate a camera. That is useful for checking planarity and parallelism of both surfaces (here performed via photo of a squeezed drop of stained water). (c) Guide pivot ensuring pure axial movement. (d) Laser meter optoNCDT 1420-50 (Micro-Epsilon, Czech Republic) with an accuracy of ± 2 μm. (e) System of weights with total mass (including the base weight) of 2798.78 g. The variance of weights mass $${m}_{i}$$ is from 46.99 g to 262.4 g. The weights were made of steel sheets with a variance of thicknesses from 0.5 to 5 mm. The weight of the individual weights was determined using an ABS320-4N (range 320 g, Kern & Sohn, Germany) laboratory scale with an accuracy of ± 0.2 mg. The mass of individual weights was written on them to avoid confusion. (f) The base weight lying on a specimen of soft tissue. The base weight consists of an aluminum plate and two linear bearings made of PTFE (colored purple in Fig. 2). Base weight mass including the bearings was 50.31 g ($${m}_{eff})$$ and its dimensions 80 × 50 mm ensured the specimen was always fully covered by it. Parallelism between base glass and base weight was verified during stand assembling via photo of a squeezed drop of stained water and was below 0.02 mm which is considered fully sufficient. (g) The 4 mm thick base glass. It is important to stress that the direct laser measurement of soft tissue thickness is unreliable^15^. Therefore we always measured the position of the top surface of the base weight instead. That is why there are holes in the additional weights (marked green in Fig. 2) so the laser beam always reflects from the top surface of the base weight.

Finally, it is noted the used base weight mass is an effective mass which means we compensated for bearings friction forces reducing the forces acting on the specimen. A spring with known stiffness k = 0.62 N/mm (measured on classical tensile test machine with 100 N load cell) was placed between the base glass and base weight. Gross base weight mass of $m=57.88 g$ should compress the spring by $w=m\cdot \frac{g}{k}=$ 0.92 mm ($g$ stands for gravity constant), while we repeatedly (5 times) measured compression of $0.80 \; \mathrm{mm}\pm 0.06 \; \mathrm{mm}$. This reduced compression was recalculated into the effective base weight mass of ${m}_{eff}=50.31g\pm 3.94g$; this value was used in all the consequent analyses. It is underlined this analysis should be always performed when the stand is assembled to check the parallelism of guiding pivots and the influence of friction.

### Methodology of measurement

The measurement began by determining the position of the top surface of the base weight without a tissue sample (see Fig. 2f) using a laser meter (Fig. 2d). This position was marked as a reference value from which the sample thickness will be determined. Next, the base weight was removed and a 30 × 30 mm sample of porcine artery excised from the anterior region of the descending thoracic aorta was placed on a glass slide (Fig. 2g). The sample was then gradually loaded by 29 weights (Fig. 2e) in ascending order. The thickness $T$ was recorded after 10 s using a laser micrometer (Fig. 2b) and radial pressure was estimated:

1 $$p=\frac{\left({m}_{eff}+\sum_{i=0}^{29}{m}_{i}\right)\cdot g}{S},$$

where $S$ is the initial area of tested sample, thus the calculated pressure corresponds to first Piola–Kirchhoff stress. Here, we take the dimensions of the cutter but our stand includes base glass (Fig. 2g) through which the sample can be photographed after being placed on the stand if a change of the initial dimensions after cutting is expected. Next, it is noted that consideration of First Piola Kirchhoff values excludes the necessity of measuring deformed sample dimensions. Finally, the sample was unloaded and immersed in a saline solution, and then placed in the refrigerator where it was stored at 5 °C until the next day. This was repeated for all ten samples.

Second analysis was performed for analyzing sample size effect. The first tested 30 × 30 mm sample was taken from the refrigerator the next morning, heated in saline solution to 37 °C for 20 min and a 20 × 20 mm sample was excised from its central region. The same measuring methodology was applied on this sample followed by nine others. The use of the same samples makes the results dependent on the sample size only and excludes the well-known inter sample thickness variability, thus. Finally, the mass of weights was converted to pressure using the cut dimensions for the next analysis.

### Unloaded thickness estimation

The obtained data were plotted in pressure vs. thickness graphs to estimate undeformed thickness. Naturally, our data are always related to a certain pressure, thus it is inevitable to extrapolate the data towards zero pressure. On the other hand, we intentionally avoid using any constitutive model to fit the data for several reasons. First, the obtained unloaded thickness would inherently depend on the chosen constitutive model while our aim is to estimate the unloaded thickness in such a way that is independent of it so it could then be used to fit any constitutive model. Secondly, application of any constitutive model would require either assumption of the loading state or its combination with finite element simulation if general loading state is expected. The first option is not realistic since the samples are very thin, thus the first guess assumption of unidirectional compression is no longer valid. A combination with FEA would remove that, but it again requires using some constitutive model. Moreover, such analysis is time consuming and can hardly be accepted as a step in the experimental protocol.

Therefore, we propose a phenomenological approach and extrapolate the data by line. This is based on our observation that the pressure-thickness response is initially linear. Naturally, this is true only in a limited pressure range, so it was critical to estimate such range. The data were therefore repeatedly fitted by a line when the number of included points gradually decreased towards zero pressure. The equation of the line reads:

2 $$T=a\cdot p+{T}_{0},$$

where $a$ is the fitting parameter and ${T}_{0}$ is the desired unloaded thickness at zero pressure. The coefficient of determination ${R}^{2}$ was gradually calculated to obtain its dependence on maximal included pressure. The ${R}^{2}$ is expected to gradually increase with lower number of included points as we are getting towards more linear area near zero pressure. At some point however the ${R}^{2}$ may reach a plateau or local maximum as we enter the linear region where noise is decisive for the ${R}^{2}$ value. Naturally, the last value is always ${R}^{2}=1$ as two points are fitted perfectly by line. We took a pressure at which the ${R}^{2}$ reached its local maximum as a limit below which the dependence can be treated as linear. Alternatively, ${R}^{2}=0.99$ was used for this limit in cases where no local maximum existed.

A line was created between points at the obtained limit pressure and the lowest pressure we had results for using (Eq. 2).This was called the 7 kPa fit approach. For comparison we also fitted a line to the three points with the lowest pressure which is further marked as “Near Zero” fit. The results were also compared statistically by non-parametric Sign test (because each 20 × 20 mm sample originated from a corresponding 30 × 30 mm sample). The null hypothesis states that there is no difference in ${T}_{0}$ between the 20 × 20 mm and 30 × 30 mm samples, whereas the alternative hypothesis states that the 20 × 20 mm samples are thinner than the 30 × 30 mm samples.

## Results

**Figure 3:**
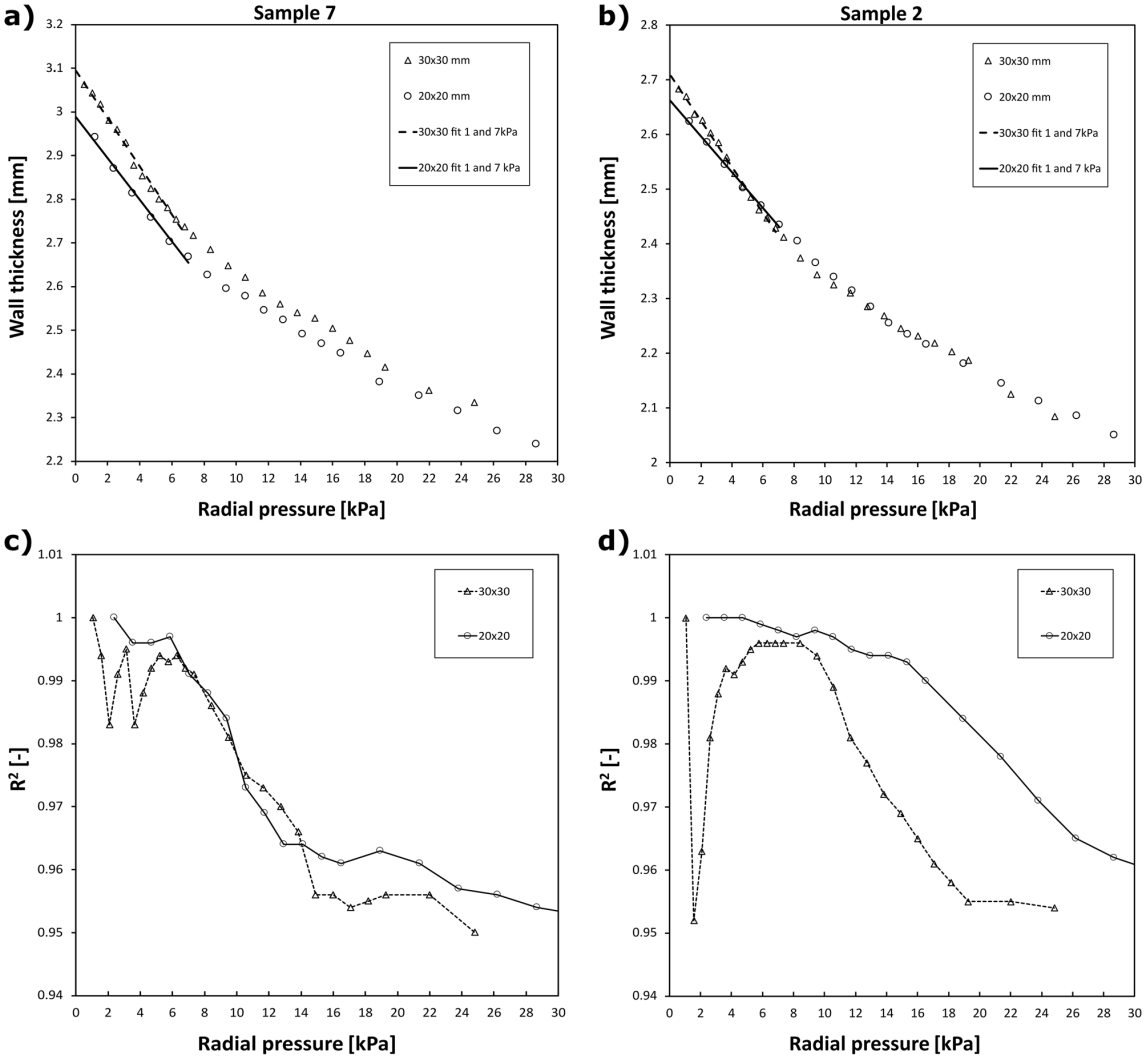
Typical variation of sample wall thickness as a consequence of applied radial pressure for samples 7 (**A**) and 2 (**B**) for two sample sizes 30 × 30 mm (triangles) and 20 × 20 mm (circles). The linear fit between 1 and 7 kPa extrapolated towards zero pressure for the 30 × 30 mm (dashed line) and 20 × 20 mm (solid line) samples. Lower row is the evolution of R^2^ values of a linear fit when gradually leaving out points with lower pressure for samples 7 (**C**) and 2 (**D**).

Overall, 20 radial compression tests were performed on 20 porcine aortic tissue samples. All measured data can be found in the supplementary data. Typical pressure-thickness responses can be seen in Fig. 3a,b. Obviously, the response is initially linear for both 20 × 20 mm and 30 × 30 mm samples. This qualitative observation was further quantified by plotting ${R}^{2}$ values of line fitting (see Fig. 3c,d). Limit pressures where ${R}^{2}$ reached either local maximum or crossed 0.99 value are shown in Table 1 for all samples. The mean limit pressure below which the response was considered linear was $6.5\pm 2.8 \; \mathrm{kPa}$ and $7.6\pm 2.7 \; \mathrm{kPa}$ for 30 × 30 mm and 20 × 20 mm samples, respectively. Out of that, we took $7 \; \mathrm{kPa}$ as a general limit pressure and constructed lines between thickness at this pressure and thickness at the highest available pressure for each sample.

**Table 1:** Radial pressure at which the R^2^ of line fitting reaches local maximum or 0.99.

Sample size	Sample no.										Mean	SD
	1	2	3	4	5	6	7	8	9	10		
	(kPa)											
30 × 30 mm	11.65	8.4	5.2	5.7	10.6	2.6	4.7	4.7	3.1	8.2	6.5	2.8
20 × 20 mm	8.2	9.4	7.4	4.7	4.7	9.4	5.9	7	4.7	14.1	7.6	2.7

The unloaded thicknesses estimated by both approaches can be seen in Table 2. The mean unloaded thickness for 30 × 30 mm samples was $2.68\pm 0.31 \; \mathrm{mm}$ and $2.68\pm 0.3 \; \mathrm{mm}$ for Near Zero fit and $7 \; \mathrm{kPa}$ fit, respectively. The mean difference in unloaded thickness estimated by both approaches was negligible: $0.01 \; \mathrm{mm}\pm 0.01 \; \mathrm{mm}$. The situation was somewhat similar for 20 × 20 mm samples. The mean unloaded thickness was $2.60\pm 0.35 \; \mathrm{mm}$ and $2.59\pm 0.35 \; \mathrm{mm}$ for Near Zero fit and $7 \; \mathrm{kPa}$ fit, respectively. The mean difference in unloaded thickness estimated by both approaches was also negligible: $0.01 \; \mathrm{mm}\pm 0.01 \; \mathrm{mm}$. Finally, both considered methods show very narrow 95% confidence intervals (CI) with respect to sample size (see Table 2).

**Table 2:** Estimated unloaded thickness for all samples. Unloaded thickness was fitted from line fitting to either the first three points or to the two points at 1 and 7 kPa. Last two columns show deviations between smaller and larger samples for both methods. CI stands for confidence interval.

Unloaded thickness ${T}_{0}$ (mm)								
Sample no.	Sample 20 × 20 mm			Sample 30 × 30 mm			Near zero fit	7 kPa fit
	Near zero fit	7 kPa fit	${\Delta }_{20\times 20}$	Near zero fit	7 kPa fit	${\Delta }_{30\times 30}$	${\Delta }_{\left(20\times 20\right)-(30\times 30)}$	
1	1.94	1.94	0.00	2.2	2.2	0.00	− 0.26	− 0.26
2	2.67	2.66	0.01	2.71	2.71	0.00	− 0.04	− 0.05
3	2.98	2.97	0.01	2.93	2.93	0.00	0.05	0.04
4	2.58	2.56	0.02	2.55	2.55	0.00	0.03	0.01
5	2.57	2.54	0.03	2.70	2.70	0.00	− 0.13	− 0.16
6	3.11	3.09	0.02	3.23	3.19	0.04	− 0.13	− 0.10
7	3.01	3	0.01	3.09	3.09	0.00	− 0.08	− 0.09
8	2.23	2.2	0.03	2.34	2.32	0.02	− 0.11	− 0.12
9	2.29	2.28	0.01	2.45	2.44	0.01	− 0.15	− 0.16
10	2.63	2.62	0.01	2.62	2.62	0.00	0.01	0.00
Mean	2.60	2.59	0.01	2.68	2.68	0.01	− 0.08	− 0.09
SD	0.35	0.35	0.01	0.31	0.30	0.01	0.09	0.09
Width of 95% CI	N/A	N/A	0.03	N/A	N/A	0.05	0.28	0.28

The median of thickness difference between smaller and larger samples is $-0.095 \; \mathrm{mm}$ for both approaches. This result, however, was not statistically significant neither for the Near Zero fit approach (p = 0.17) nor for the 7 kPa fit approach (p = 0.09). Generally, two possible behavior of samples was observed as shown in Fig. 3. Either combination of various loads and sample sizes marked the same pressure-thickness curve (see Sample 2 in Fig. 3) or the smaller sample marked visibly different pressure-thickness curve (see Sample 7 in Fig. 3).

## Discussion

In this study, we proposed a general approach on how to measure unloaded wall thickness of soft tissues. In order to do that, we designed and manufactured a simple force-driven experimental stand (see Fig. 2). Our intension was designing a stand which can be placed and operated next to classical tensile test machine rather than designing stand within such machine (i.e. using load cell, deformation tracking tools etc.). It is because such (expensive) machine is typically used for performing the tensile tests and switching its setup back and forth from thickness measurement to tensile testing would be very time consuming or even impossible in cases when saline solution bath is used for testing^2,13^. Our design can be further modified, i.e. the laser micrometer can be replaced by a regular camera (positioned perpendicularly to the stand) or any other contactless measurement device possessed by researchers. We, however, warn from using of weights without guiding as we experience severe tilting of the weights on sample when guiding pivots (see Fig. 2c) were omitted. It resulted in non-repeatable measurements especially in area of larger pressures.

The proposed approach relies on linear regression and it is operator independent and applicable to any soft tissues. It does not require neither any constitutive modelling nor finite element simulations. Performed analyses confirmed the validity of the most critical assumption that pressure-thickness response is linear near zero pressure (Fig. 3a,b) and we also estimated a limit pressure to which the linearity holds ($7 \; \mathrm{kPa}$) for porcine aorta prepared as described in “Tissue preparation”. Knowing that, we can perform further testing much faster since only two (or three if noise should be considered) weights resulting in pressures below $7 \; \mathrm{kPa}$ can be used; therefore, single sample thickness can be estimated in less than a minute. Furthermore, the fact that there were no significant differences in wall thicknesses between smaller and larger samples also means that the combination of local thickness heterogeneity, time of smaller samples storage, changes of dimensions after cutting and intra operator variability is negligible in the reported method. We acknowledge, however, that similar analysis should be performed once for every tested type of soft tissue and every different way of tissue preservation method (i.e. fresh vs. frozen tissue) as this may not be the general rule.

Unloaded thickness estimated by our approach is naturally larger than values reported by others (see Table 3 for who tested the same tissue). Although these values can be compared only qualitatively, the systematic shift caused by using a measurement method generating some (often undefined) contact pressure is obvious. Differences in wall thickness estimation are one of the major sources of differences between reported mechanical responses (both elastic and failure) of soft tissues^15,28^.

**Table 3:** Mean or median porcine descending thoracic aorta as reported by various studies.

Study	Thickness measurement method	Mean/median of porcine thoracic aortic wall thickness (mm)
Sigaeva et al.^13^	Caliper (unknown contact pressure)	2.1–2.2 (both anterior)
Polzer et al.^2^	Thickness gauge 1 kPa contact pressure	2.31 (anterior)
Peňa et al.^23^	Digital micrometer 0.5 N (unknown contact pressure)	2.51
Kim and Baek^24^	Caliper (unknown contact pressure)	1.5(anterior) 2.2(posterior)
Stemper et al.^25^	Caliper (unknown contact pressure)	1.8
Peňa et al.^26^	Digital micrometer 0.5 N Contact pressure 10 kPa or 13 kPa	2.46
Delagadillo et al.^27^	Caliper (unknown contact pressure)	2.3

Proposed method is also sufficiently accurate. The standard deviation in base weight mass caused by friction (and other measurement features) is only 3.94 g and its importance is further diminished by the fact it uses points calculated with mass several times exceeding the base weight mass. Additional weights are not affected by friction as they lay on the base weight freely, thus they do not add to this uncertainty. Instead they decrease the relative error (see Fig. 2). As it can be checked in the supplementary data, the base weight change by at least 4 g or 15 g is necessary to affect the unloaded wall thickness by 0.01 mm for the 20 × 20 mm and 30 × 30 mm large samples respectively. Therefore, under assumption of error being normal distributed we can conclude our method uncertainty is 0.02 mm and 0.01 mm for 20 × 20 mm and 30 × 30 mm sample size respectively. That is fully comparable with other methods such as caliper or micrometer measurements^15^. Moreover, obtained widths of 95% CI (0.28 mm for both methods, see Table 2) are only about 40% of those obtained by thickness gauge which is considered the best tool for nondestructive arterial wall thickness measurement so far^15^.

Sample size was not observed to affect the measured wall thickness. Although these results should be treated with discretion since the p-values are relatively low and number of samples is not very high, it is expected result. The thickness is primarily measured here so the sample size (of uniformly thick material) should not affect the measured thickness. The same weights create different pressures only when applied on smaller samples, thus marking different points on the same pressure-thickness curve. That was observed in some cases (see Sample 2, in Fig. 3 and Supplementary Data) yet more commonly we observed decrease of the pressure-thickness curve for smaller sample (see Sample 7 in Fig. 3 and Supplementary Data). This can be explained for instance by water outflow as the cutting edges allow the inner liquid to flow out and the cutting surfaces are relatively larger with respect to sample volume for smaller samples. Further investigation is however needed on this topic.

Despite promising results, it is important also to mention the limitations of our study. First of all, it is the viscoelastic nature of the soft tissues which meant that we had to choose a time scale at which the wall thickness is measured. The choice of 10 s is an arbitrary trade-off value between instantaneous and long-term response. Shorter times would be more suitable for simulation of in-vivo response while longer times would better correspond with quasi-static tensile tests at which mechanical properties are often measured^2,13,23^. A second limitation can be seen in the 24-h time delay between in measurements of larger and smaller samples. The experimental protocol could be rearranged in future to test smaller samples right after the larger one. Nevertheless, no measurable effect on estimated wall thickness was noticed after 24 h in refrigerator which is in line with other study^25^ so possible improvement would be somewhat minor. Finally, it is noted the sample dimensions after cutting may not be the same as dimensions of the cutter due to release of residual stresses. If that is a concern, the sample initial area S in Eq. (1) should be taken from a photograph. Nevertheless, such changes affect only the estimated radial pressure but not the wall thickness which is measured directly.

## Conclusions

In this study, we developed a new procedure for measuring the unloaded thickness of soft biological tissue samples. It is based on linear extrapolation of the experimentally measured pressure-thickness response towards zero pressure. The proposed method does not rely on any assumptions of constitutive models or loading states. In addition, the application of this method can remove systematic shifts in reported mechanical properties of soft tissues caused by the use of contact thickness measurement methods. Uncertainty of the proposed method (0.03 mm for 20 × 20 mm sample) is comparable to other devices while the width of 95% CI is only about 40% of those reported with the best different tool (thickness gauge).

## Supplementary Information

Supplementary Information.

## Data Availability

All data generated or analyzed during this study are included in this published article and its supplementary information files.

## References

[1] Sacks MS, Chuong CJ (1993). Biaxial mechanical properties of passive right ventricular free wall myocardium. J. Biomech. Eng..

[2] Polzer S (2015). Structure-based constitutive model can accurately predict planar biaxial properties of aortic wall tissue. Acta Biomater..

[3] Gusic RJ, Petko M, Myung R, Gaynor JW, Gooch KJ (2005). Mechanical properties of native and ex vivo remodeled porcine saphenous veins. J. Biomech..

[4] Horny L (2012). Age-related distribution of longitudinal pre-strain in abdominal aorta with emphasis on forensic application. Forensic Sci. Int..

[5] Tsamis A, Krawiec JT, Vorp DA (2013). Elastin and collagen fibre microstructure of the human aorta in ageing and disease: A review. J. R. Soc. Interface.

[6] Maher E (2009). Tensile and compressive properties of fresh human carotid atherosclerotic plaques. J. Biomech..

[7] Bruder L, Pelisek J, Eckstein HH, Gee MW (2020). Biomechanical rupture risk assessment of abdominal aortic aneurysms using clinical data: A patient-specific, probabilistic framework and comparative case-control study. PLoS ONE.

[8] Vitasek R, Gossiho D, Polzer S (2020). Sources of inconsistency in mean mechanical response of abdominal aortic aneurysm tissue. J. Mech. Behav. Biomed. Mater..

[9] Sigaeva T, Sattari S, Polzer S, Appoo JJ, Di Martino ES (2021). Biomechanical properties of ascending aortic aneurysms: Quantification of inter- and intra-patient variability. J. Biomech..

[10] Lally C, Dolan F, Prendergast PJ (2005). Cardiovascular stent design and vessel stresses: A finite element analysis. J. Biomech..

[11] Walsh MT (2014). Uniaxial tensile testing approaches for characterisation of atherosclerotic plaques. J. Biomech..

[12] Acosta Santamaría VA, Flechas García M, Molimard J, Avril S (2018). Three-dimensional full-field strain measurements across a whole porcine aorta subjected to tensile loading using optical coherence tomography—Digital volume correlation. Front. Mech. Eng..

[13] Sigaeva T, Polzer S, Vitásek R, di Martino ES (2020). Effect of testing conditions on the mechanical response of aortic tissues from planar biaxial experiments: Loading protocol and specimen side. J. Mech. Behav. Biomed. Mater..

[14] Slazansky M, Polzer S, Man V, Bursa J (2016). Analysis of accuracy of biaxial tests based on their computational simulations. Strain.

[15] O’Leary SA, Doyle BJ, McGloughlin TM (2013). Comparison of methods used to measure the thickness of soft tissues and their influence on the evaluation of tensile stress. J. Biomech..

[16] Di Martino ES (2006). Biomechanical properties of ruptured versus electively repaired abdominal aortic aneurysm wall tissue. J. Vasc. Surg..

[17] Raghavan ML, Webster MW, Vorp DA (1996). Ex vivo biomechanical behavior of abdominal aortic aneurysm: Assessment using a new mathematical model. Ann. Biomed. Eng..

[18] Reeps C (2013). Measuring and modeling patient-specific distributions of material properties in abdominal aortic aneurysm wall. Biomech. Model Mechanobiol..

[19] Nolan DR, McGarry JP (2016). On the compressibility of arterial tissue. Ann. Biomed. Eng..

[20] Kermani G, Hemmasizadeh A, Assari S, Autieri M, Darvish K (2017). Investigation of inhomogeneous and anisotropic material behavior of porcine thoracic aorta using nano-indentation tests. J. Mech. Behav. Biomed. Mater..

[21] Chuong CJ, Fung YC (1984). Compressibility and constitutive equation of arterial wall in radial compression experiments. J. Biomech..

[22] O’Leary SA, Doyle BJ, McGloughlin TM (2014). The impact of long term freezing on the mechanical properties of porcine aortic tissue. J. Mech. Behav. Biomed. Mater..

[23] Peña JA, Martínez MA, Peña E (2019). Failure damage mechanical properties of thoracic and abdominal porcine aorta layers and related constitutive modeling: phenomenological and microstructural approach. Biomech. Model. Mechanobiol..

[24] Kim J, Baek S (2011). Circumferential variations of mechanical behavior of the porcine thoracic aorta during the inflation test. J. Biomech..

[25] Stemper BD (2007). Mechanics of fresh, refrigerated, and frozen arterial tissue. J. Surg. Res..

[26] Peña JA, Martínez MA, Peña E (2015). Layer-specific residual deformations and uniaxial and biaxial mechanical properties of thoracic porcine aorta. J. Mech. Behav. Biomed. Mater..

[27] Virues Delgadillo JO, Delorme S, El-Ayoubi R, DiRaddo R, Hatzikiriakos SG (2010). Effect of freezing on the passive mechanical properties of arterial samples. J. Biomed. Sci. Eng..

[28] Polzer S (2020). Failure properties of abdominal aortic aneurysm tissue are orientation dependent. J. Mech. Behav. Biomed. Mater..

